# Chronic Diffuse Sclerosing Osteomyelitis of the Mandible: The Use of Bisphosphonates as a Treatment for a Rare and Challenging Condition

**DOI:** 10.3390/medicina60060917

**Published:** 2024-05-30

**Authors:** Edgar Keller, Susanna Clocchiatti, Katia Rupel, Giulia Ottaviani, Chiara Ratti, Gianluca Canton, Roberto Di Lenarda, Luigi Murena, Matteo Biasotto

**Affiliations:** 1 Department of Medical, Surgical and Health Sciences, University of Trieste, 34100 Trieste, Italy; kelleredgar93@gmail.com (E.K.); krupel@units.it (K.R.); rdilenarda@units.it (R.D.L.); m.biasotto@fmc.units.it (M.B.); 2 Clinica Ortopedica e Traumatologica, Ospedale di Cattinara, Azienda Sanitaria Universitaria Giuliano Isontina (ASUGI), 34100 Trieste, Italy; susanna.clocchiatti@libero.it (S.C.); chiara.ratti@asugi.sanita.fvg.it (C.R.); gcanton@units.it (G.C.); lmurena@units.it (L.M.)

**Keywords:** antiresorptive therapy, bisphosphonates, CDSO, sclerosing osteomyelitis

## Abstract

Chronic diffuse sclerosing osteomyelitis is a very rare condition, described as a non-suppurative, inflammatory disease of the bone and characterized by a proliferative endosteal reaction, which clinically reveals itself with cyclic pain of the jaw and swelling. We reported two clinical cases, where patients suffered recurrent swelling and pain at the mandible irradiating to the preauricular area, denying any previous trauma or significant medical history. Odontogenic causes were excluded. An initial treatment with antibiotics and NSAIDs temporarily relieved the symptoms without complete resolution, prompting further investigations. After a comprehensive array of diagnostic tools (X-rays, CT scans, scintigraphy, bone biopsy, serum markers), both patients were diagnosed with chronic diffuse sclerosing osteomyelitis of the mandible. Bisphosphonates (clodronate and zolendronate) with different treatment schemes were used to treat the condition, until a full recovery from symptoms was reported. Bisphosphonates could therefore represent an effective option in managing this rare but impactful condition. Further research is warranted to better understand the underlying mechanisms of the disease and to optimize treatment strategies.

## 1. Introduction

Osteomyelitis defines a group of diseases that are characterized by an inflammatory process of the entire bone, beginning in the medullar cavity and then extending to the cortex and periosteum. Etiological factors primarily include infections by pyogenic microorganisms, but also trauma, radiation, and chemical substances. The classification system by Hudson divides different forms of osteomyelitis into two major categories, namely, acute (suppurative and non-suppurative) and chronic osteomyelitis [1]. 

Among the different chronic forms, “chronic diffuse sclerosing osteomyelitis (CDSO)” [2] is a specific form of non-suppurative osteomyelitis that is characterized by recurrent long-term pain and swelling, usually in the mandible, with radiographic features including sclerosis, a periosteal reaction, osteolysis, sclerosis of the mandibular canal, and widening of the lamina dura. It was described by Jacobsson [3] in 1984 as a distinguished clinical entity, and the prevalence is estimated to be 1:200,000. Due to its rarity and challenging recognition, it has been described with different and confusing nomenclatures.

CDSO has no specifically accepted diagnostic criteria, and its diagnosis relies on a combination of a patient’s medical history and clinical, radiographic, and histological findings. It is sustained by an endosteal proliferative reaction, which expresses itself with a cyclic clinical history consisting of periods that are free of symptoms followed by periods where the patient reports swelling and pain without a clear acute onset, and therefore, it is considered a primary form of osteomyelitis [4]. Clinical features include recurrent swelling, intense pain, trismus and progressive mandibular asymmetry without abscesses, internal or external fistula, or sequestrum formation. Serum markers of inflammation are usually high, while bone turnover markers are within normal ranges, contributing to a differential diagnosis with osseous dysplasias. Radiographic signs include sclerosis, subperiosteal bone formation, a periosteal reaction, a loss of demarcation between the cortical and medullary bone, and mandibular canal boundary loss. The bone histology describes sclerosis without cellular abnormalities, often with inflammatory infiltrates. Notably, CDSO typically shows a marked scintigraphic uptake of the affected regions [3].

The diagnosis is challenging and is often made based on exclusion; differential diagnoses include Chronic Recurrent Multifocal Osteomyelitis (CRMO) [5], Paget’s disease of bone [6], and Osteosclerotic bone dysplasia (mielorheostosis) [7]. CRMO is multifocal, with uncommon mandibular involvement, and is associated by some authors to the SAPHO syndrome, which is characterized by synovitis, acne, pustulosis, hyperostosis, and osteitis [8]. Paget’s disease of bone may present in monostotic forms showing bone asymmetry and augmented scintigraphic uptake, but its distinctive features are alteration of bone turnover markers (especially bone-specific alkaline phosphatase) and histologically abnormal bone remodeling [6]. Osteosclerotic bone dysplasia is characterized by abnormal bone deposition with multiple focal areas of osteosclerosis, which shows slow progression without pain and scintigraphic uptake [7].

Moreover, the symptoms of CDSO often resemble those of odontogenic infections, making it crucial for general dentists to perform proper clinical and instrumental evaluations to exclude such conditions and recognize the diagnosis. This ensures a timely initiation of treatment, which is especially important considering that a delay in diagnosis and improper therapies are possibly leading not only to severe physical, psychological, and social repercussions but also to substantial economic costs related to healthcare assistance and a loss of personal productivity, as well as an overall decline in the quality of life of patients suffering from such severe pain.

Due to deceiving nomenclature and the rarity of the disease, there is a lack of clinical trials and, therefore, no standard treatment protocols. Treatment options include medical therapy with antibiotics and corticosteroids or surgical approaches, which show limited long-term efficacy [9]. Some studies reported the efficacy of bisphosphonates, with the complete resolution of symptoms [10]. These drugs have a well-known antiresorptive activity, primarily targeting osteoclasts and thus acting in areas affected by a high bone turnover. Considering that bisphosphonates represent a major risk factor for developing Medication-Related Osteonecrosis of the Jaws (MRONJ), an extensive dental evaluation is mandatory prior to their prescription in order to identify and remove local risk factors [11,12]. 

## 2. Case Report

### 2.1. Patient 1

A 42-year-old man presented to the Dental Emergency Unit (Maxillofacial surgery and Dental Clinic, Ospedale Maggiore, Trieste), reporting recurrent left hemifacial pain and swelling, which had previously been treated with antibiotic therapy without improvement. The pain irradiated from the left mandible to the maxilla and the preauricular area, with an intensity of 8 out of 10 on a Numerical Rating Scale (NRS) [13]. The patient reported that the swelling initially improved after the systemic antibiotic therapy was initiated (amoxicillin/clavulanate), although it did not completely recover. There was no history of local trauma, and remote and family pathological history were negative. The patient was taking NSAIDs (ibuprofene, ketorolac) both during daytime and during the night to ease the pain and discomfort.

**Figure 1:**
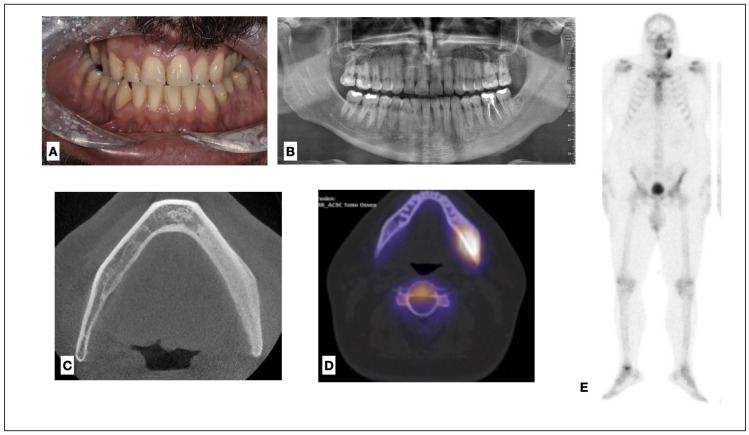
Intraoral view (**A**). Dental panoramic radiograph: a slight increase in the bone density of the left hemimandible can be noted (**B**). CT scan in axial view of the mandible, with marked sclerosis of the left hemimandible (**C**). Axial view of the scintigraphy, with a marked hyperuptake of the left mandible (**D**). The total body scintigraphy does not show any other areas of involvement (**E**).

Extraoral and intraoral examinations revealed no abnormalities of the cutaneous mucosal tissues (Figure 1A) and mouth-opening movements showed no restrictions. The periodontal health of his teeth was assessed through periodontal probing, and all teeth in the area were found to be vital after thermic testing. A slight pain on percussion was revealed on tooth number 36. A dental panoramic radiography revealed unilateral sclerotic appearance of the left mandible (Figure 1B). 

Because of the minimal pain on percussion and the slight symptomatic remission with antibiotics, an initial diagnosis of acute apical abscess was made, and endodontic therapy of tooth 36 was performed. A gnathological examination including TMJ function and palpation of masticatory muscles was also performed by an expert gnathologist, and a putative diagnosis of myofascial pain was suggested. Consequently, an inferior bite and myorelaxants were prescribed. After one month, the patient did not feel any relief from the pain and was still using NSAIDs daily. 

Subsequently, the patient was re-evaluated, and additional diagnostic tests were performed: bone biopsy, scintigraphy, a CT scan, and specific blood tests (calcium, alkaline phosphatase, bone alkaline phosphatase, vitamin D 25-OH, parathyroid hormone, inorganic phosphate, C-terminal telopeptide, osteocalcin, erythrocyte sedimentation rate, and C-reactive protein).

The histological examination revealed small bone tissue fragments, mostly fragmented, dystrophic, and involuted, mixed with areas of amorphous and hemorrhagic material. No infiltrative vital cellular elements were highlighted, and no areas of altered bone remodeling were detected.

The erythrocyte sedimentation rate was high (29 mm/h), whereas C-reactive protein was normal (0.6 mg/dL). Total leukocytes were within normal limits, as were the bone metabolism markers (calcium, alkaline phosphatase, vitamin D, parathyroid hormone, and inorganic phosphate). Paget’s disease and osteomyelitis were therefore excluded from differential diagnoses.

A CT scan (Figure 1C) detected an extended endosteal sclerosis, with cortical thickening and moderate periosteal bone formation of the left mandible, which extended from the canine region to the mandible ramus, without condylar resorption.

Scintigraphy with Tc99HMDP revealed marked hyperuptake of the osteotropic tracer in the left hemimandible and high osteo-metabolic activity in the left hemimandible, which was compatible with altered bone remodeling (Figure 1D,E). The mandible was the only affected area.

Based on all testing and clinical evidence, a diagnosis of CDSO was suggested. Firstly, intramuscular clodronate therapy was initiated (400 mg/mL every 2 days for 2 weeks, then 400 mg/mL weekly for 6 weeks). The patient pointed out an improvement in symptoms, but pain was still present a few months after the interruption of clodronate therapy (7 out of 10 on NRS scale) [8].

Subsequently, intravenous amino bisphosphonate therapy was prescribed; a single injection of Zoledronic Acid (5 mg in 100 mL saline solution 0.9%) was administered, and a complete resolution of symptoms was observed within one week.

At 15 months after the single-dose injection, the patient reported a pain intensity of 3 out of 10 (NRS), with a need to take NSAIDs two times per month in the last 2 months). Another single injection of Zoledronic Acid was performed 20 months after the first administration. The patient reported a complete resolution of symptoms.

### 2.2. Patient 2

**Figure 2:**
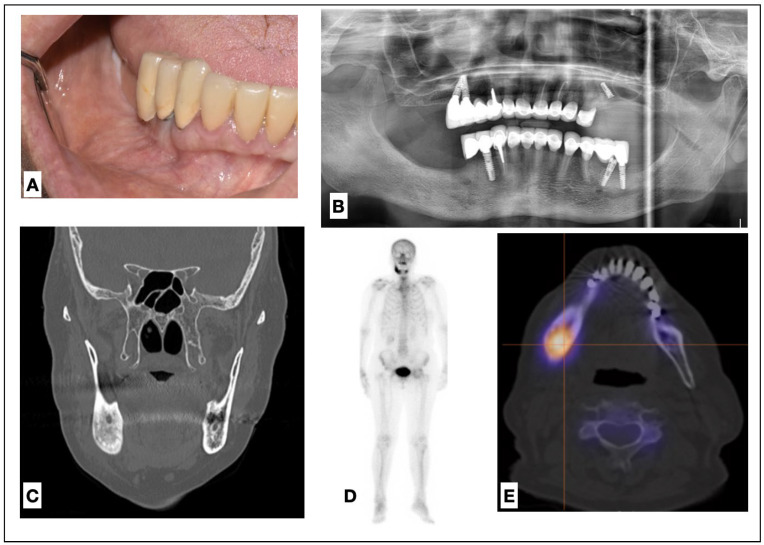
Intraoral view, no significant alteration can be noticed (**A**); dental panoramic radiograph, a slight increase in bone density of the right hemimandible can be noticed (**B**); CT scan in coronal view revealing endosteal sclerosis of the left hemimandible (**C**); total body scintigraphy showing a marked hyperuptake of the right mandible; no other area of involvement was found (**D**); axial view of scintigraphy with a marked hyperuptake of the left mandible (**E**).

A 72-year-old woman presented to the Oral Medicine and Pathology Unit (Maxillofacial surgery and Dental Clinic, Ospedale Maggiore, Trieste) complaining of pain of the right hemimandible, 7 out of 10 on the NRS Scale [13]. The pain had been present for 2 months, during which the patient was taking NSAIDs daily. An intraoral examination revealed partial edentulism and multiple fixed prosthetic rehabilitations (Figure 1A). An accurate exam of the dental, implant, and periodontal tissues, as well as dental panoramic radiograph, excluded any odontogenic conditions (Figure 2B) but revealed marked sclerosis of the left hemimandible.

Subsequently, a bone biopsy, blood tests (blood count and C-reactive protein), a CT scan, and a scintigraphy with Tc99HMDP were prescribed. The histopathological examination revealed the presence of compact bone tissue, characterized by marginal necrotic phenomena and extensive areas of fibrotization, without signs of altered or dysplastic bone remodeling. Blood tests including markers of inflammation and bone turnover, which were within normal limits. CT scans revealed endosteal sclerosis of the right hemimandible and a ground glass appearance of the bone marrow (Figure 2C), while the scintigraphy (Figure 2D,E) revealed a marked hyperuptake of the osteotropic tracer in the right hemimandible. 

A diagnosis of CDSO was made, and the patient was administered oral clodronate therapy with a dosage of 400 mg every 2 days for 2 weeks, and 400 mg weekly for 6 weeks. The patient reported a marked improvement of symptoms after this treatment, without recurrence at 1 year follow-up. 

## 3. Discussion

CDSO is a rare and challenging condition. Patients usually present with recurrent swelling and pain of the mandible; a progressive deformity and trismus may also be present [10], and condylar resorption has also been reported [4].

The etiology remains unclear, and various hypotheses have been postulated, which include infectious triggers, the manifestation of autoimmune diseases, or systemic syndromes such as synovitis, acne, Pustolosis, hyperostosis and osteitis syndrome (SAPHO), and Chronic Recurrent Multifocal Osteomyelitis (CRMO) [14,15,16,17]. Chronic tendoperiostitis as a result of hyperactivity of the masticatory muscles has also been proposed as a trigger for CDSO [18,19].

As a consequence, many different treatments have been suggested, including surgery, antibiotic therapy, anti-inflammatory drugs, antiresorptive drugs, conservative treatment, and hyperbaric oxygen therapy. In the review by van de Meent et al., it was concluded that surgical and hyperbaric oxygen therapies had a low rate of success in the management of the condition. Conservative treatment and antiresorptive drugs were found to be more successful and should be considered as first-line treatments in patients affected by CDSO. Bisphosphonates showed a success rate of 94% in improving patients’ discomfort; half of these showed complete remission of symptoms, while some patients underwent a second injection. This could possibly be explained by the inflammatory response that is possibly triggered by an increased bone turnover. The reduction in bone turnover caused by antiresorptive therapy therefore reduces inflammation and connected swelling and pain. Different types of bisphosphonates have been suggested, both nitrogen-containing bisphosphonates (pamidronate, alendronate, ibandronate, and zoledronate) and nitrogen-free bisphosphonates (disodium clodronate). Long-term side effects such as MRONJ have not been reported yet [9]. In a case report by Jerkovic et al., two patients affected by CDSO were treated with denosumab; both patients found benefit from the single injections, but each administration was less successful than the previous in terms of pain reduction and the duration of the effect [20].

In the only double-blinded randomized controlled trial published so far, the authors compared the administration of intravenous disodium clodronate therapy with intravenous placebo. The results showed a significant reduction in pain after 6 months in patients receiving clodronate therapy, but no significant differences were found after 12 months [21]. 

Our findings were in line with these results: Patient 2 had a complete resolution of symptoms after clodronate therapy, while Patient 1 found partial relief after the same treatment. Intravenous Zoledronic Acid therapy was then chosen to try to achieve pain cessation. 

**Figure 3:**
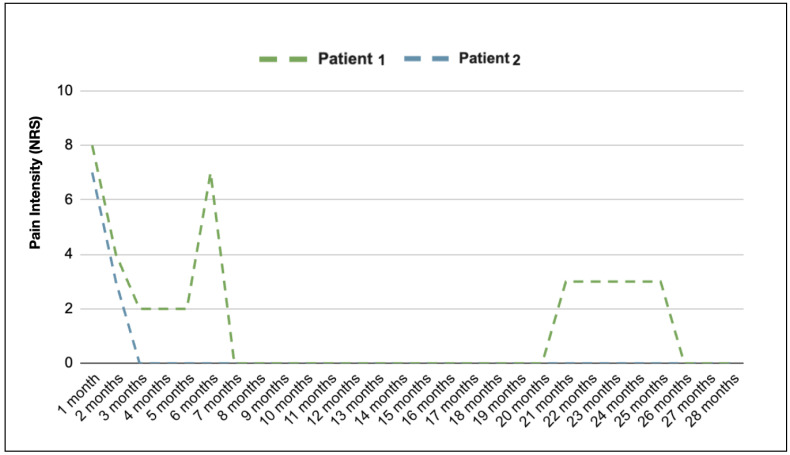
**Patient 1** had a slight resolution of symptoms 3 months after clodronate therapy; symptoms were still present 2 months after the interruption of therapy, and intravenous Zoledronic Acid was initiated. Despite a complete resolution of symptoms being achieved, the pain worsened 15 months after the first injection. A second injection was administered, and a complete resolution of symptoms was observed. **Patient 2** had a complete resolution of symptoms after clodronate therapy, and a complete resolution of symptoms was reported 3 months after therapy administration.

Disodium clodronate exhibits a lower affinity for bone, resulting in its eventual depletion, and is characterized by a late-onset effect. The antiresorptive potencies of the nitrogenous compounds are higher than those of clodronate, leading to an extended period of efficacy [22]. This may explain the different results obtained in terms of the duration of pain relief in Patient 1. In our cases, Figure 3 depicts how the pain intensity decreased immediately after the antiresorptive drug administration in both patients. This suggests that bisphosphonates could emerge as a promising first-line treatment option for patients with CDSO.

## 4. Conclusions

CDSO poses significant challenges in both diagnosis and management, underscoring the need for effective therapeutic strategies. Our experience suggests that bisphosphonates could emerge as a promising first-line treatment option to mitigate symptoms and enhance patient outcomes. Moreover, deepening the mechanisms through which bisphosphonates alleviate symptoms may offer valuable insights into the elusive nature of this disease. Further research efforts aimed at elucidating the intricate interplay between bisphosphonates and CDSO’s pathology are needed to offer higher treatment efficacy of this challenging condition.

## Data Availability

All data are available upon request to the corresponding author.
